# Supplementary material for: Are carbon emissions trading and green financial instruments synergistic? -Comprehensive quantitative research based on content analysis
Source: PLoS One. 2024 Mar 7;19(3):e0298601. doi: 10.1371/journal.pone.0298601 (PMC10919601; doi:10.1371/journal.pone.0298601)
Supplement: S1 Data — (ZIP) [file pone.0298601.s001.zip › Data/地级市Z_F工作报告.zip!/╔┬╬≈/╤╙░▓╩╨/└┤╫╘╣1⁄2╓┌║┼í╛┬φ┐╦╩2╛▌═°í┐_2016.docx]

各位代表：

现在，我代表市人民政府向大会报告工作，请予审议，并请市政协委员和其他列席人员提出意见。

一、2015年和“十二五”工作回顾

2015年，是我市进入新世纪以来经济下行压力最大、发展最为艰难的一年。面对严峻形势，我们以习总书记回延安视察重要指示为动力，全力稳增长、调结构、促改革、惠民生、防风险，埋头苦干，迎难而上，经济总体呈现缓中趋稳、稳中向好态势。完成生产总值1198.63亿元，增长1.7%；全社会固定资产投资1637.17亿元，增长6.2%；地方财政收入161.17亿元，下降4.1%；城镇居民人均可支配收入33127元，增长8.3%；农民人均纯收入10775元，增长10.2%。

（一）全力稳增长促投资。针对上半年国际油价断崖式下跌、油煤产量下降、主要经济指标负增长的被动局面，及时出台稳定工业、促进投资、发展服务业、扩大招商引资和增收节支等五方面52条政策措施，强化经济运行调度，落实重大项目专项推进和领导干部帮扶重点企业责任制，支持企业稳产促销，生产总值、固定资产投资降幅逐季收窄并实现扭负为正。原油生产加工、煤炭产量基本稳定，天然气产量增长89.6%，卷烟产量增长4.2%，苹果、蔬菜、肉蛋产量持续增长，来延游客和旅游综合收入分别增长11.3%和12.1%。市级重点项目完成投资852亿元，增长10.9%。稳增长、促投资等系列措施落实受到国务院督查表扬。

（二）大力推进经济结构调整。黄陵煤矸石电厂、黄龙风电等一批项目建成投产，大唐热电联产、富县煤油气资源综合利用、深圳轻工产业园区等项目建设进度加快，陕建安塞新型材料工业园区等一批非能工业项目开工建设。签订招商引资合同项目269个，到位资金620.3亿元，增长21.2%。与阿里巴巴、彩虹集团、西京公司等企业在大数据、光伏产业等领域合作项目加快推进。累计建成现代农业示范园区40个，新增省市农业龙头企业20家、农民专业合作社253个。洛川50万亩绿色苹果基地通过国家验收。26个重大文化旅游项目快速推进，枣园文化广场、文安驿文化产业园区、乾坤湾景区、黄陵国家森林公园等项目建成运营。电子商务交易额增长65%。举办了革命老区电商发展峰会，“延安大礼包”上线销售。新增“四上”企业197户、市场主体2.5万户。全社会消费品零售总额增长10.5%。建筑业增加值增长12.5%。第三产业和非公经济占GDP比重分别达到28.8%和23.4%，较上年分别提高6.9个和2.3个百分点。

（三）全面提升四级城镇承载服务能力。开展城市建设百日会战活动，新区4条连接老城道路和13条市政道路建成通车，3所学校建成投用，市级行政中心、市民服务中心基本建成，延安大剧院、市中医院、6所幼儿园和部分市民住房主体封顶。城市核心区居民下山、建筑物外立面改造、七里铺、尹家沟等棚户区改造和清凉山、宝塔山山体综合治理基本完成。扎实推进“2+1”三城联创，市容环境持续改善,通过了省级生态园林城市验收。县城承载能力明显增强，黄陵、黄龙被命名为国家园林县城。重点镇、新型农村社区完成投资82.2亿元，服务功能不断完善，产业园区加快建设。5.06万农民进城落户，城镇化率达到57.3%。

（四）持续改善生态环境和基础设施条件。造林绿化80.6万亩，治沟造地11.3万亩。市区公交车、出租车、客运车辆、市政用车全部实现油改气，淘汰黄标车和老旧机动车7280辆，实施锅炉煤改气11台，新增集中供热面积20万平方米，城区空气质量优良天数达到282天，比上年增加30天。完成延川、甘泉、宜川、黄陵4个县城污水厂提标改造。四项主要污染物减排指标全面完成。农村环境集中连片整治成效显著。延延高速和黄延高速扩能工程西安至富县段建成通车，吴定高速、蒙华铁路开工建设。

（五）加大精准扶贫力度。认真贯彻落实陕甘宁革命老区脱贫致富座谈会议精神，出台了《脱贫致富行动方案》和《精准扶贫实施意见》，全面打响脱贫攻坚战。全市7.62万户、20.52万贫困人口全部建档立卡，落实移民搬迁、产业培育、能力提升、整村推进、救助兜底、包村联户“六大扶贫措施”，3.7万名干部驻村帮扶，启动5万户、17.5万人移民搬迁，154个移民搬迁安置点全部开工建设，建成安置房3.9万套，搬迁入住9319户、32617人。8.83万贫困群众实现脱贫，黄龙、吴起、志丹3县整体脱贫。

（六）扎实办好民生实事。新增城镇就业1.9万人，城镇登记失业率3.57%，农村劳动力转移就业31.5万人。城乡居民医疗、养老保险实现全员参保，提高了医保补贴、基础养老金标准和企业退休人员基本养老金，为17.3万城乡低保对象发放低保金3.24亿元。完成机关事业单位工资调整。黄陵、吴起实现省级教育强县目标，安塞、宜川实现“双高双普”,延川、安塞、富县通过国家义务教育发展基本均衡县评估认定。宜川中学被确定为市县共建区域性重点中学。我市被国家确定为城市公立医院综合改革试点，延安医疗集团总医院对宜川县医院实现托管。圆满完成第十一届中国艺术节火种采集。第四代歌剧《白毛女》在延安成功首演。安塞县被授予“中国曲艺之乡”。

（七）深入推进各项改革。启动35大项、203子项年度改革任务，完成188项。3家银行在延设立分支机构，全市银行存贷款余额首次突破2000亿元,贷款余额增长22.2%。全年融资到位107亿元。全面完成农村土地承包经营权登记颁证，流转土地50.5万亩。启动自然资源资产负债表编制试点。全市精减政府机构388个。建立了事业单位临聘人员管理制度。完成撤乡并镇和市级行政机关公车改革。启动首批6户国企改制。

（八）提高政府自身建设和社会治理水平。扎实开展“三严三实”专题教育，持续推进“四风”整治。全面实行全口径预算管理，市级“三公”经费支出下降32.3%。清理下放行政审批事项66项，精简率33.9%。自觉接受人大、政协和社会各界监督，办理人大代表建议117件、政协委员提案419件，向市人大报告专项工作17次，接受专题询问2次，与市政协协商了养老服务业发展、清真餐饮业管理等工作。公民代表走进政府、大学生到政府机关见习成为常态。办理网民留言168件，办结率100%。妥善化解信访积案374件，5个县实现“四无”创建目标。安全生产四项指标全面下降，被省政府授予全省安全生产先进市。社会治安满意率提高到93.15%。

国防动员、防灾减灾、民族宗教、质量监督、食品药品、外事侨务、新闻出版、文史社科、妇女儿童、残疾人、人防、体育、气象、档案、地方志等工作取得了新的成绩。

各位代表，总结2015年，困难比预料的多，结果比预想的好，实现了“十二五”圆满收官。回顾“十二五”发展历程，全市上下坚持以统筹城乡发展为总揽，大力发扬功成不必在我和低调务实不张扬的作风，扎扎实实调结构、转方式，打基础、利长远，经济社会发展取得重大成就。

五年来，我们始终坚持发展第一要务，主动适应经济发展新常态，经济综合实力大幅跃升。经济总量、全社会固定资产投资先后突破千亿元大关，生产总值比“十一五”末增长35.4%；全社会固定资产投资总量是前五年的2.8倍。地方财政收入比“十一五”末增长53.2%。城镇居民人均可支配收入、农民人均纯收入超过全国全省平均水平，年均分别增长13.1%和15.8%。

五年来，我们始终坚持把经济结构调整作为首要任务，狠抓园区建设、项目支撑、集群推进，转型发展动力显著增强。“三区九园”工业布局初步形成，入园企业334户。延安能源化工集团、民营投资集团等一批助推转型发展的新兴力量加速成长。建成183个重大能源转化和非能源项目。粮食实现十二连丰，苹果种植面积居全国地级市之首，省级优质苹果基地县实现整市全覆盖，被国家命名为现代农业示范区。完成国家级洛川苹果批发市场一期工程建设。建成18个重大文化旅游项目，接待游客、旅游综合收入分别比“十一五”末增长1.4倍和1.5倍。完成企业登记制度和“三证合一”改革，市场主体突破10万户。全社会消费品零售总额增长106%。第三产业、非公经济占GDP比重分别提高8.6个和6.3个百分点。三次产业结构由“十一五”末的8:71.8:20.2调整为9.2:62:28.8。

五年来，我们始终坚持以人的城镇化为目标，全面加快四级城镇体系建设，新型城镇化快速推进。实施“中疏外扩、上山建城”战略，新区开创了延安城市建设新纪元，必将成为带动经济社会发展的新引擎。老城改造推动城市面貌发生全新变化，10万人搬离山体窑洞迁入新居，一大批市政设施项目建成投用，城市功能显著改善，人居环境持续优化，圣地形象进一步彰显。12个县城建成区面积扩大42%，35个重点镇有31个基本成型，100个新型农村社区有56个达到建设标准。累计有29.3万农民进城落户，城镇化率提高9个百分点。

五年来，我们始终坚持生态优先，加快生态环境和基础设施建设，支撑发展的条件明显改善。自筹资金完成新一轮退耕还林160.2万亩。治理水土流失面积5100平方公里，治沟造地35.6万亩。城市空气质量稳居全省前列。被列为首批全国生态文明先行示范区和第二批低碳城市试点。“三纵两横”高速公路网加快建设，新增高速公路288公里，增加铁路客运班次23趟，开通了延安至西安、宝鸡动车。延安新机场开工建设，增加城市航线3条，立体交通网络正在形成。南沟门水利枢纽工程实现南线供水，黄河引水全面开工。57.8万农民实现安全饮水。电力、通信保障能力不断提升。

五年来，我们始终坚持保障和改善民生，大力实施惠民工程，人民群众获得感进一步提升。累计民生投入1117.1亿元，是“十一五”的2.3倍。45.78万人搬出深山，32.98万贫困人口实现脱贫。全面完成特大强降雨灾后恢复重建，7.4万房屋倒损户搬入新居，工农业生产和基础设施恢复到灾前水平。率先在全省实现居民养老保险、医疗保险制度城乡一体化。公立医院改革走在全省前列，组建了延安医疗集团，完成了对4家医院托管。率先在全省实现学前三年免费教育和义务教育营养改善计划全覆盖，实施了城区教育资源整合，择校热、上学难、大班额问题有效缓解。保障房建设走在全省前列，8.48万户住房困难群众入住新居。文化事业繁荣发展，推出了《延安颂》、《延安保育院》等剧目并保持常态化演出，“延安过大年”被评为全国群众文化活动“群星奖”，一批民间艺术家将陕北文化推向全国。“六五”普法通过省上验收。先后蝉联全国双拥模范城“七连冠”和全省双拥模范城“八连冠”。

各位代表，回首过去的五年，全市干部群众为圣地延安、生态延安、幸福延安建设贡献了聪明才智，付出了艰辛努力，成绩来之不易，经验弥足珍贵。这是市委坚强领导的结果，是市人大、市政协支持监督的结果，是全市上下开拓创新、攻坚克难、奋力拼搏的结果。在此，我代表市人民政府，向奋战在全市各条战线的广大干部群众、中省驻延单位、驻延部队指战员、武警官兵和公安民警致以崇高的敬意！向各位人大代表、各位政协委员、各民主党派、工商联和社会各界人士表示衷心的感谢！

在看到成绩的同时，我们也清醒地认识到，我市发展中还存在一些突出矛盾和问题。一是经济结构不合理，新的发展动力不足，在油煤量价齐跌双重因素影响下，长期积累的各种矛盾集中显现，稳增长、调结构、保运转的压力持续加大；二是区域和城乡发展不平衡，基本公共服务供给不足，精准脱贫任务艰巨，民生保障还有不少薄弱环节；三是资源环境约束趋紧，基础设施欠账较多，发展保障能力还需进一步提高；四是创新社会治理、优化发展环境、提升行政效能的任务依然艰巨，依法行政意识有待增强；五是干部中作风不严不实的问题仍然存在，政府系统党风廉政建设还需进一步加强。对这些问题，我们将高度重视，认真扎实解决，努力把各项工作做得更好，不辜负全市人民的期望和重托。

二、“十三五”基本思路和目标任务

“十三五”是我市实现率先脱贫、全面建成小康社会的决胜期，是全面深化改革的攻坚期，更是我市推进结构调整和转型发展的关键期。按照中省和市委要求，我们编制了“十三五”规划纲要草案，提出了未来五年发展目标、主要任务和重大举措，本次会议审议批准后，我们将认真组织实施。

“十三五”的主要预期目标是：生产总值年均增长7%左右，全社会固定资产投资年均增长15%，地方财政收入年均增长7%，城乡居民收入年均分别增长10%和12%，“十三五”末生产总值、城乡居民收入比2010年翻一番，2018年率先在全国革命老区中实现脱贫，2020年与全国人民同步够格进入全面小康社会。

各位代表，确定这样一个目标，是全面建成小康社会的需要，是全市人民的共同期盼，是我们肩负的重大责任和庄严使命。当前和今后一个时期，国家“一带一路”、加大脱贫攻坚、支持革命老区开发建设等一系列重大战略和政策的实施，为我们发挥后发优势、争取中省支持带来了新的机遇；十八届五中全会提出创新、协调、绿色、开放、共享五大新的发展理念，全面部署推进供给侧结构性改革，对我市加快调结构促转型、推进新型城镇化、补齐发展短板提供了新的空间；经过多年的扎实打基础，全市发展思路更加明晰，基础要素条件、环境保障和金融支撑能力显著提升，全面深化改革的红利正在释放，全市上下人心思进、共谋发展的氛围更加浓厚。我们将按照“四个全面”的战略布局，进一步增强使命感、责任感和紧迫感，坚定发展信心，努力追赶超越，以奋发有为的精神状态，投身全面建成小康社会的伟大事业！

今后五年，重点在五个方面实现突破发展：

坚持创新发展，加快产业体系重塑，培育转型发展新动能。以“三区九园”为主要承载平台，把创新驱动作为结构调整、转型发展的第一动力，加强科技合作交流与成果转化，围绕发展高端能化、绿色载能和新兴产业，加快建设石油、煤炭、天然气和煤电“四大基地”，推进煤电一体化，加快1500万千瓦火电基地和电力外送通道建设，打造千亿级能源产业集群；推进一批油煤气资源深度转化重大项目，打造500亿级能化产业集群；大力发展新型复合陶瓷、多晶硅、新材料等产业，打造绿色载能产业集群；培育发展新兴产业，建设500万千瓦新能源基地，打造新能源、装备制造、大数据三大百亿级新兴产业集群。推进国家现代农业示范区建设，构建新型农业经营体系，加快建设果品、小杂粮等农产品加工基地，打造百亿级农产品精深加工产业集群。构筑全域旅游新格局，大力发展红色旅游、黄帝历史文化旅游、黄河自然遗产旅游、黄土风情文化旅游和绿色生态旅游，打造五大文化旅游产业板块。加快发展现代物流、电子商务等新型业态，改造提升传统服务业，打造百亿级现代物流产业集群。

坚持协调发展，加快新型城镇化步伐，构筑区域一体发展新格局。以延河、洛河、秀延河、黄河沿岸为轴带，包茂、榆蓝、青兰、延吴高速公路为廊道，构建“四带四廊”空间布局，引导产业配置，带动城镇发展。按照中央城市工作会议“五个统筹”要求，加快中心城市建设，推进新区和老城规划建设管理，建设宜居宜业宜游城市，建成全国文明城市和国家卫生城市。加快安塞、甘泉撤县设区和子长、黄陵撤县设市，形成“一主两副”为主干的区域性中心城市格局。继续推进县城、重点镇和新型农村社区建设，增强人口集聚、产业承载和辐射带动功能，全面提升四级城镇体系建设水平，城镇化率达到65%以上。加快构建布局合理、互联互通、城乡共享的基础设施网络，打造区域性综合交通枢纽，消除水资源瓶颈，提升电力保障能力，构建覆盖城乡、服务便捷的信息网络。

坚持绿色发展，加强生态文明建设，厚植持续发展新优势。围绕建设全国生态文明先行示范区，全面实施主体功能区规划。加快推进“陕北高原大绿化”，实施1000万亩长青生态林建设，巩固提升退耕还林成果。全面完成延河综合治理，实施防洪保安、水土保持、水资源配置、水环境治理、水生态建设五大任务，努力把延河建成堤固、洪畅、水清、岸绿、景美的老区人民幸福河。完成治沟造地50万亩。大力发展循环经济，推进园区循环化改造和废旧资源回收再利用。加强饮用水源地保护，全面推行排污许可，加快城镇污水、垃圾处理设施建设，稳步提升主要河流水质。严守耕地保护红线，节约集约用地。继续加强大气污染治理，持续提升城区空气质量。建成国家森林城市、生态园林城市、节水型城市、环保模范城市，努力使延安的天更蓝、山更绿、水更清、空气更清新。

坚持开放发展，破解制约难题，催生发展新活力。以问题为导向，深化行政管理、财税管理和农村综合改革，推动金融服务创新，全面完成国有企业改革，进一步释放发展活力。推进大众创业、万众创新，培育形成一批“顶天立地”的企业集团和“铺天盖地”的中小微企业。主动融入“一带一路”，加强与呼包银榆经济区、关天经济区的交流合作，实现互利多赢、共同发展。加大招商引资力度，围绕绿色载能、精细化工、装备制造、高技术产业、新材料等重点领域，主动承接产业转移。加快发展口岸贸易、跨境电商、服务外包等外向型经济。编制人才队伍建设中长期规划，大力培养重点产业、重要领域急需人才。完善人才引进激励机制，不拘一格引进技术创新、经营管理等紧缺实用人才。鼓励高等院校、科研院所、大型企业在延设立重点学科和实验室，培养一批高素质技能型专门人才。

坚持共享发展，增加公共服务供给，推动民生保障再上新水平。按照社会政策托底要求和普惠性、保基本、均等化、可持续方向，不断提升公共服务能力，让更多发展成果惠及广大群众。坚决打赢脱贫攻坚战，2018年贫困人口全部脱贫，贫困县区全部摘帽，让贫困人口都能同步够格进入小康。实施促进创业带动就业五年行动计划，推动重点人群多渠道就业，让每个家庭都能有稳定可靠收入。健全完善社会保障体系，推进社会福利由补缺型向适度普惠型转变。坚持教育优先发展，深化教育改革，促进教育公平和质量提升，让所有孩子都能接受良好教育。加快公共卫生服务体系建设，完成城市公立医院综合改革试点任务，继续推动优质医疗资源下沉，让人人都能看得上病、看得起病。大力发展文化产业，繁荣文化事业，用社会主义核心价值观凝聚共识、汇聚力量。

三、2016年主要工作

今年是我市调结构促转型、推进精准脱贫的关键一年。我们将扎实推进供给侧结构性改革，认真落实“去产能、去库存、去杠杆、降成本、补短板”五项重点任务，努力实现“十三五”良好开局。主要预期目标是：生产总值增长6%左右，全社会固定资产投资增长10%，地方财政收入下降19.3%，城乡居民收入分别增长9%和10%。

（一）全力稳增长调结构，保持经济平稳运行

打好“三去一降一补”歼灭战。支持延长集团根据能源市场变化和企业经营实际，寻找新的产销平衡点。扩大天然气生产，产量达到55亿立方米，实现以气补油。加快煤炭资源整合，稳定煤炭产量。大力支持新“延安”牌卷烟生产销售，推动非能工业持续增长。全年规上工业增加值增长4.8%。分类推进国企改革，加快处置42户“僵尸企业”，年内完成6户企业改制任务。打好降本增效组合拳，清理减免面向中小企业收费，实施涉企收费目录清单管理。积极为企业提供融资担保支持，帮助置换高息民间贷款，降低企业成本。落实房地产去库存政策措施，下功夫化解商品房库存，推动房地产业健康发展。

力促工业转型升级。坚持“以转促去”消化过剩产能，推动油煤气资源深度转化。开工永炼油煤混炼扩能改造、子长500万吨煤炭高温干馏气化综合循环利用、陕煤黄陵电厂、延长石油富县电厂、洛川轻烃裂解综合利用、安塞20万吨LNG等项目，加快富县煤油气资源综合利用、延长石油伴生气综合利用、大唐热电联产、华能电厂和风电、光伏发电项目建设，促进富县双翼石化煤焦油深加工等项目达产达效。开工西京多晶硅、彩虹光伏玻璃、能新科装备制造等项目，加快经开区产业孵化基地、深圳轻工产业园区、陕建安塞新型材料工业园区及PC构件、延川龙基医药生产基地等项目建设。

促进现代农业提质增效。加快洛川苹果、甘泉禽豆制品等8个农产品加工园区建设，重点扶持一批农产品加工企业做大做强。新启动13个现代农业示范园区建设，以园区带动特色农产品基地建设。继续推动苹果北扩，新增苹果面积10万亩。举办第九届中国·陕西(洛川)国际苹果博览会。出台扶持发展新型农业经营主体的政策措施，大力培育更多龙头企业、专业合作社、家庭农场和加工养殖大户。加强洛川苹果、延川红枣、延安小米、延安地椒羊肉、黄龙核桃等优势农产品品牌管理和保护，继续推进农业标准化生产和农产品质量认证体系、可追溯体系建设，不断提升品牌知名度和市场占有率。加快81个淘宝村建设，鼓励发展订单农业和农产品线上线下销售，促进农产品产销快捷对接。健全农业防灾减灾体系，提高自然灾害防范能力。

推动文化旅游产业加快发展。以26个重大文化旅游项目为引领，扶持发展一批文化旅游龙头企业，系统打造特色文化旅游品牌。完成宝塔山、凤凰山、枣园旧址保护提升和圣地河谷金延安板块、中国革命艺术家博物院、黄帝文化中心主体工程，开工建设延安博物馆、北京知青文化展览馆、陕甘宁边区革命英烈纪念馆、黄河壶口文化景区、黄帝陵景区旅游服务中心、黄陵秦直道古城等项目，加快恢复抗大等重要革命旧址，推动延安红色系列景区、壶口瀑布创建国家5A级旅游景区。建成黄帝陵门户网站，开展网上祭祖活动。加快推进文安驿、甘谷驿、安定、双龙、冯庄、直罗、南泥湾等文化旅游名镇建设。启动实施一批森林探险游、生态避暑游、乡村体验游和冬季冰雪运动等旅游项目。大力发展红色教育培训产业，培训3.5万人次。加快发展智慧旅游，实现重点景区景点公共无线网络全覆盖，启动红色旅游大数据平台建设。以纪念红军长征胜利80周年和举办第十一届中国文化艺术节开幕式为契机，精心策划系列文化旅游宣传营销活动。持续开展旅游环境综合整治，开通旅游景区公交专线，完善旅游配套设施，形成服务旅游、支持旅游大环境，促进游客和旅游综合收入持续增长。

支持现代服务业突破发展。出台扶持电子商务发展的政策措施，大力培育电商龙头企业和具有延安特色的电商品牌，引导电商与制造、农业、商贸、仓储、物流、快递等产业融合发展。加快构建生产装备、建材家居、农产品、小商品四大物流体系，启动中国供销农产品物流园区、富县煤炭物流园区建设，加快安塞物流产业园区、吴起德胜物流园区、洛川现代苹果产业物流园区建设，支持延安利源等一批物流企业迅速成长。积极发展金融服务、文化休闲、体育健身、养老托幼等消费业态，支持服务主体围绕消费新需求增加有效供给，建设城市商业聚集区和“15分钟”社区便民圈。继续抓好“四上”企业培育，加快推进个转企。

扶持非公有制经济快速发展。启动扶持中小企业发展五年行动计划，带动中小企业快速成长。支持民营企业和科研院所合作，提高中小企业创新能力和产品质量，引导中小企业走“专、精、特、新”的发展路子。鼓励中小企业组建经济联合体和实业集团，进入能源化工下游产业、装备制造、商业零售及新兴产业领域。完善市县中小企业服务平台功能，强化融资、用地等方面服务保障和政策支持，鼓励金融机构开发适宜中小企业特点的金融产品，出台支持大学生到中小企业就业的政策措施，有效破解企业资金、人才短缺难题。

（二）着力促投资补短板，持续改善基础设施和生态环境

努力促进投资持续增长。紧扣中省政策取向，进一步提高投资的有效性、精准性，积极引进企业、基金加快“三区九园”和县域工业园区建设，完善基础设施和公共服务配套，促进更多项目进入园区。启动宝塔区青化砭能源化工园区和方塔新兴产业园区建设。落实重大项目建设责任，强化土地供应、资金筹措、资源配置等要素保障，推动项目加快实施。加强项目谋划论证，发挥低电价、低气价等比较优势，加大招商引资力度，吸引更多企业和项目落户。

加大基础设施建设力度。黄延高速扩能工程、沿黄公路年内建成通车，加快推进延安新机场、蒙华铁路、包西高铁、吴定高速建设，开工绥德至延川高速、蒲城至黄龙高速、延安北过境和一批县城过境线,力争开工清涧至子长高速。实施乡村道路改造提升工程。南沟门水利枢纽工程实现全面供水，黄河引水工程黄延线全线贯通，启动引洛济延工程，继续推进一批县城、重点镇供水工程建设。积极推行阶梯水价、分质供水和中水回用，促进水资源高效利用。加快国家A类城市配电网和电力输送通道建设，实施一批330、110千伏输变电工程。推进智慧城市建设，积极争取全国电信骨干节点城市政策，尽快实现延安与全国骨干节点城市宽带信息通道连接，推动大数据产业基地建设。

加强生态建设和环境保护。坚持抓好天然林保护、三北防护林、桥山保护、秦晋峡谷绿化、湿地公园、森林公园等重点项目建设。坚持封山禁牧，巩固退耕还林成果。启动国有林场改革。完成治沟造地8万亩。进一步落实大气、土壤、水源等污染防治措施，继续推进城镇锅炉煤改气和环保煤使用，持续开展农村环境集中连片整治，全面推进延河综合治理，实施中心城区段和安塞、延长县城段河道整治、堤防建设等十大项目。支持宝塔区实施丰富川、杜甫川等城市周边片区综合整治。坚持开展油气开发安全综合治理，努力实现油气管道隐患全面清零目标，坚决遏制污染事故发生。加快编制全市自然资源资产负债表，实行能源、水资源、建设用地总量和强度双控制度。开展自然资源资产党政领导干部离任审计试点，严格生态环境损害责任追究，从源头上防止浪费资源、污染环境、破坏生态。

（三）加快四级城镇体系建设，推进新型城镇化

继续推进中心城市建设改造。实施城市建设“十大工程”，新区市级行政中心、市民服务中心5月底前投入使用，延安大剧院和干部职工住房、保障房、安置房按期交付，建成桥沟、东十里铺2条新老城连接线和一批道路，启动石油博物馆、历史博物馆、全民体育运动中心、2所九年制义务学校和一批公园、商业设施建设。加快岩土尾留工程建设。继续推进以棚户区改造为重点的老城改造，启动宝塔山周边、长青路山体、大砭沟沟道等棚改项目，加快南门坡周边和七里铺片区改造，完成主城区山体居民下山。统筹推进海绵城市、综合管廊、灯光景观、生态绿化等重大项目，完善水电气暖、垃圾污水处理等基础设施和公共服务，新建一批公园、广场，不断提升城市功能和形象。

进一步提升城市管理水平。全面推行“多规合一”，出台城市建设规划导则，完善中心城市设计。严格规划管理，切实维护规划严肃性。理顺市容环境管理体制，夯实街道办和社区监管职责，加强城市管理综合执法。深入推进“2+1”三城联创，持续开展市容市貌、环境卫生、广告牌匾、建筑工地、经营秩序等专项整治，加快城市道路环线、停车场、过街设施、公厕、便民市场等建设，优先发展公共交通，着力解决出行难、停车难、如厕难、买菜卖菜难等城市病问题。广泛开展文明市民教育，不断提高市民素质。加强居民小区物业管理，全面提升公共服务能力。

加快县城、重点镇和新型农村社区建设。制定县城、重点镇、新型农村社区建设规划指导意见，科学确定县城发展定位，加快产业园区、基础设施配套、棚户区改造等项目建设。争取完成安塞撤县设区。继续推进35个重点镇和100个新型农村社区建设，按规划标准完善基础设施、公共服务和产业配套，增强承载带动能力。全面推行居住证制度，加快消除进城落户农民政策待遇和社会保障差异，推进城镇基本公共服务常住人口全覆盖，促进农业转移人口市民化。

（四）全面深化各项改革，破解体制机制制约

打好国企改革攻坚战。以处置停产半停产、长期亏损、资不抵债或靠财政补贴、银行续贷存活的企业为重点，通过兼并、重组、破产等方式，盘活国有资产，妥善安置困难企业职工。大力发展混合所有制经济，鼓励民营企业通过参股、控股、资产收购等形式参与国有企业改制，增强企业活力。改进国有资产监管体制，推动生产性企业健全现代企业制度，完善法人治理结构，做强做大做优一批国有企业。

继续深化投融资改革。加快各类基金设立，深化与北京紫马财行、光大证券等金融机构合作。积极引进金融业总部机构、分支机构及新型金融业态落户延安，支持金融机构延伸服务网络，创新金融产品。全力支持能源集团、城投公司、旅游集团等企业通过并购、上市等方式提高竞争实力。大力推动政府和社会资本合作，推出一批PPP项目，吸引社会资本参与基础设施建设。

大力推进财政管理改革。树立长期过紧日子思想，坚持量入为出、以收定支、厉行节约，优化支出结构。加强财政专项资金管理，全面推行零基预算和绩效评价，实行部门事业费、专项经费项目化管理，实现绩效目标全覆盖。市县共同压减财政支出，建立财政保基本稳定调节基金，提高县域间对口帮扶资金筹集比例。完善协税护税工作机制，加大税收征管力度，努力实现应收尽收。

加快行政审批改革。加强行政审批事项动态管理，完善基本建设项目并联审批机制，切实把“放、管、服”改革推向深入。加快商事制度改革，探索推行“证照分离”，推进营业执照和企业登记全程电子化。推行企业信用监管体系建设，建立失信受限多部门联合惩戒机制。积极推广“互联网+政府服务”模式，全面实行投资项目审批监管线上运行，实现实体政务大厅服务向网上办事服务延伸。改进直接面向企业和群众的公共服务，坚持推行乡村两级群众办事干部代办制度。

（五）加大脱贫攻坚力度，确保实现年度脱贫目标

扎实推进精准扶贫。按照“六个精准”和“五个一批”要求，持续加大扶贫投入，大力推行龙头企业与合作社带动发展产业、移民搬迁、金融扶持、生态旅游、劳务输出、社保兜底等扶贫模式。建立脱贫攻坚长效机制，完善精准识别、精准扶贫台账，实行有进有出的动态管理。夯实县乡主体责任和干部帮扶责任，积极争取央企、省企和民营企业开展帮扶贫困村活动，构建专项扶贫、行业扶贫、社会扶贫互为补充的大扶贫格局。年内实现8.7万贫困人口和宝塔、安塞、子长、甘泉4县区整体脱贫。

全力抓好扶贫移民搬迁工程。完善易地扶贫搬迁规划，核实搬迁对象，明确安置方式和迁入选址。用好国家易地扶贫搬迁补助资金，用活贫困老区城乡建设用地指标增减挂钩省域流转政策，整合交通建设、农田水利、土地整治、地质灾害防治、林业生态等涉农资金和社会资金，加快搬迁安置点公共服务设施配套。今年启动6.25万贫困人口易地扶贫搬迁，力争用两年时间全面完成12.5万建档立卡贫困人口搬迁。继续推进避灾移民搬迁。

完善兜底保障政策措施。实现农村低保标准与扶贫标准“两线合一”。建立困难残疾人生活补贴和重度残疾人护理补贴制度，对低保家庭中的老年人、未成年人、重度残疾人等重点救助对象按照低保标准增发补助金。制定实施农村贫困人口慢性病医疗救助办法，有效解决因病致贫返贫问题。对贫困家庭学生实行从学前、小学、中学、大学直至就业“一条龙”帮扶。对无法依靠产业扶持和就业帮助脱贫的家庭实行政策性保障兜底。重视城市困难群体生活，多渠道解决贫困人口就业问题。

（六）扎实抓好民生工程，提升群众生活水平

坚持以创业促就业。全面落实推动大众创业万众创新实施意见，加大创业担保贷款、贴息贷款发放力度，加强众创孵化体系和创新创业基地建设。完善培训促就业机制，支持延安职业技术学院开展产学结合、校企合作，围绕产业集群和园区建设，加强技术工人、电商和新型职业农民等实用人才培训，促进重点人群就业。落实就业补贴政策，鼓励企业吸纳就业，新增自主创业1万人、城镇就业1.5万人，城镇登记失业率控制在3.9%以内。健全完善劳务输出对接机制，劳务输出27万人次。

完善社会保障体系。进一步完善城乡居民基本医疗保险制度和大病保险制度，探索推行总额预付、单病种付费、多种付费方式和智能控费管理机制，实行差异化报销。积极推进生育保险市级统筹。依法推进各类用人单位参加工伤保险。全面推行居家养老、社区服务养老服务模式，扶持建设社会经营性敬老院。落实好农村“三留守”人员、空巢老人等弱势群体的救助保障措施。新开工保障性住房2.8万套、建成1.3万套。结合房地产去库存，加快建立购租并举的住房保障制度，打通商品房转为保障性住房、移民搬迁安置房通道，满足新市民住房需求。

促进教育全面发展。继续推进城区校际联盟和学区制管理，完善校际联盟考核机制。科学调整中小学布局，优化教育资源配置，加快实施县城以上学校建设改造任务，积极推进重点镇学校标准化建设。继续实施第二期学前教育三年行动计划，加快公办幼儿园建设，公办幼儿园和在园幼儿数量均达到50%以上。加强学校管理和教师队伍建设，扎实推进素质教育，促进教育内涵式发展。启动延安大学迁建工程。支持延安职业技术学院争取进入陕西省高水平示范性高职院校行列，全面启动创建国内一流高职院校建设计划。加强校园周边环境治理，保障学生身心健康和安全。

加快卫生计生事业发展。完成延安医疗集团县级分院对重点镇卫生院的一体化管理，全面推行分级诊疗。推进城市公立医院综合改革试点和市人民医院“创三甲”工作，完成市中医院建设，启动市妇女儿童医院、口腔专科医院建设和市精神病专科医院、传染病专科医院改扩建，推进乡镇卫生院规范化创建，加强地方病、传染病防治。发挥延大医学院作用，加强基层全科医生和医护人员培训。落实好一对夫妇可生育两个孩子政策。

丰富群众文化体育生活。加快推进陕北文化生态保护实验区建设，建立非物质文化遗产数字化档案库。创新政府购买公共文化产品机制，大力支持文艺创作，推出一批具有影响力的文学、影视、戏剧和民间艺术精品。支持安塞申办“中国民歌节”。加快基层综合性文化服务中心建设，新建一批文化室、文化大院，继续开展“三秦书月•书香延安”全民阅读活动。加强文化市场监管，净化文化市场环境。加快普及校园足球，完善青少年体育俱乐部制度。继续实施全民健身工程，推进体育健身器材进重点镇、进社区。

全面加强社会治理。深入排查化解矛盾纠纷，加强网络舆情研判、引导和处置，强化信访案件督查督办，扎实解决群众反映强烈的突出问题。落实各级安全监管责任和企业主体责任，突出抓好道路交通、公众消防、非煤矿山、危化品、特种设备等重点行业领域安全监管，坚决遏制重特大安全事故。坚持开展食品安全示范县创建活动，突出小餐饮、小摊贩和小食品店整治，全面提升食品药品安全保障水平。继续推进平安延安建设，完善社会治安防控体系，依法打击“两抢一盗”、黄赌毒、非法集资、电信诈骗等违法犯罪活动，不断增强人民群众安全感，争创全国综治优秀市。启动实施“七五”普法，深入开展“法律六进”，增强全民法治观念。强化民族宗教事务管理。深入开展“双拥”共建，巩固军政军民团结。

四、加强政府自身建设

以推进依法行政转变政府职能。上半年公布市县两级政府部门权力清单、责任清单、专项资金管理清单和企业投资项目负面清单，做到法无授权不可为、法定职责必须为。建立政府起草地方性法规和政府规章制定程序，积极推进地方立法工作。健全依法决策机制，把公众参与、专家论证、风险评估、合法性前置审查、集体讨论决定作为政府重大决策法定程序，提高科学决策水平和公共决策透明度。坚持和完善向人大报告工作、与政协协商工作、向老干部和各界人士通报工作制度，自觉接受各方面监督。推进行政执法改革，细化量化行政裁量基准，建立行政执法全过程记录制度，做到严格、规范、公正、文明执法。完善政务公开制度，坚持政府新闻发布制度，依法向社会公开涉及公共利益、公众权益和社会关注事项，扩大行政权力运行、公共资源交易等领域信息公开，及时回应社会关切。

以开展“两学一做”学习教育加强作风建设。巩固群众路线教育实践成果，落实“三严三实”专题教育整改事项，严格执行中央八项规定和国务院“约法三章”要求，深化“四风”突出问题治理，持之以恒抓好作风建设。严格控制行政成本，继续精简会议文件，保持“三公”经费只减不增。完善群众和服务对象监督评价部门及窗口单位制度，坚决纠正行政不作为、慢作为、乱作为等行为。加强政务服务中心建设，优化政府门户网站，认真办理市民热线和网民留言，做到事事有回音、件件有着落。

以强化责任担当提升工作效能。牢固树立责任意识、忧患意识，认真尽责履职，主动作为，奋发有为。强化决策落实机制，继续推行服务承诺、限时办结等制度，完善绩效管理和考核评价体系，推进重点工作、重大事项落实。创新导向激励机制，推进干部“能上能下”，切实解决“为官不为”问题。探索建立合理容错机制，尊重和保护干部干事创业的积极性。健全跟踪督查机制，强化行政效能监察，进一步提高政府执行力和公信力。

以严格监督问责促进廉政建设。坚决贯彻全面从严治党要求，自觉遵守《廉洁自律准则》和《纪律处分条例》，带头强化党性修养和规矩意识，严格履行党风廉政建设主体责任，全面落实“一岗双责”。坚持用制度管权管事管人，加强领导干部执行公务接待、因公出国境、住房和办公用房、个人重大事项报告等相关制度的监督检查，实现公共资金、国有资产、国有资源和领导干部履行经济责任审计监督全覆盖，严肃查处违规违纪行为，以实际行动赢得人民的信任和支持。

各位代表，宏伟蓝图鼓舞人心，发展目标催人奋进。让我们以习总书记回延安视察重要讲话精神为指引，在省委、省政府和市委的坚强领导下，团结依靠全市人民，继承和发扬延安精神，切实担当起引领新常态、推动新发展的历史重任，凝心聚力，砥砺奋进，为我市实现率先脱贫、全面建成小康社会目标而努力奋斗！
